# Study of Auditory Brain Cognition Laws-Based Recognition Method of Automobile Sound Quality

**DOI:** 10.3389/fnhum.2021.663049

**Published:** 2021-10-08

**Authors:** Liping Xie, Chihua Lu, Zhien Liu, Lirong Yan, Tao Xu

**Affiliations:** ^1^Hubei Key Laboratory of Advanced Technology for Automotive Components, Wuhan University of Technology, Wuhan, China; ^2^Foshan Xianhu Laboratory of the Advanced Energy Science and Technology Guangdong Laboratory, Foshan, China

**Keywords:** automobile sound quality, EEG, brain cognition laws, Kalman smoothing, mRMR

## Abstract

The research shows that subjective feelings of people, such as emotions and fatigue, can be objectively reflected by electroencephalography (EEG) physiological signals Thus, an evaluation method based on EEG, which is used to explore auditory brain cognition laws, is introduced in this study. The brain cognition laws are summarized by analyzing the EEG power topographic map under the stimulation of three kinds of automobile sound, namely, quality of comfort, powerfulness, and acceleration. Then, the EEG features of the subjects are classified through a machine learning algorithm, by which the recognition of diversified automobile sound is realized. In addition, the Kalman smoothing and minimal redundancy maximal relevance (mRMR) algorithm is used to improve the recognition accuracy. The results show that there are differences in the neural characteristics of diversified automobile sound quality, with a positive correlation between EEG energy and sound intensity. Furthermore, by using the Kalman smoothing and mRMR algorithm, recognition accuracy is improved, and the amount of calculation is reduced. The novel idea and method to explore the cognitive laws of automobile sound quality from the field of brain-computer interface technology are provided in this study.

## Introduction

Methods that are applied to evaluate automobile sound quality mainly rely on the psychological feelings of people and cannot guarantee the universality of evaluation results (Tan and Tan, 2012). Methods of ranking, semantic differentiation (Guo et al., 2017), grade score, pairing comparison (Parizet, 2002; Ellermeier et al., 2004) are commonly used for subjective evaluation. However, when the sound qualities with similar semantics (such as “comfort,” “powerfulness,” and “acceleration”) are designed under the dominance of sound forward design, and the traditional subjective evaluation methods are difficult to reflect the true feelings of the evaluator. In addition to inherent physical parameter characteristics of sounds, the evaluation of an evaluator for the sound is also related to their cognition, experience, and emotional state (Genuit, 2004). Therefore, it is necessary to introduce a new automobile sound quality evaluation method for evaluating the diversified automobile sound.

### Related Works

In recent years, with the research on physiological signals in emotional computing, it has become possible to use physiological signals to evaluate automobile sound. EEG signals with high time and spatial resolution are widely used (Lin et al., 2010; Bhatti et al., 2016; Geethanjali et al., 2018).

The analysis of EEG signals is challenging, and the analysis of EEG signals in the field of emotion recognition relies on data pre-processing, feature extraction (Tsang et al., 2010; Kai et al., 2016; Poikonen et al., 2016), and feature classification. Feature extraction is crucial to ensure recognition performance. Only by selecting EEG features closely related to the purpose of research can effectively meet the performance of recognition (Nishimura and Mitsukura, 2013; Sheykhivand et al., 2020). Some studies indicated that rhythm characteristic of EEG can reflect human brain activities, which are δ (1–4 Hz), θ (4–8 Hz), α (8–12 Hz), β (12–30 Hz), and γ (>30 Hz) (Knyazev, 2012; Zheng and Lu, 2015). Chen et al. (2021) proposed an EEG physiological acoustic index to evaluate subjective annoyance by comparing EEG rhythm characteristics and the change in the trend of subjective annoyance index data. Li et al. (2014) used white noise and pure tone as stimulus sources to study the relationship between EEG characteristic signals and subjective annoyance, and it is found that the average power of θ waves has two peaks in each brain area during steady stimulation. Ali et al. (2013) studied EEG signals under different sound pressure levels and stimulation intervals, and the study found that the θ wave voltage increased significantly because of high sound pressure level stimulation. Di and Wu (2015) showed that the average α wave power in the left frontal lobe was significantly lower than that in the right frontal lobe under the stimulation of pleasant sounds.

In the study of automotive sound quality and EEG signals, Lee and Lee (2014) introduced a new method to study human sound perception by means of EEGs, where EEG analysis and measurement were performed to demonstrate human cerebral response to car acceleration sounds and concluded that the α-wave power could serve as an objective evaluation index of automobile acceleration sounds. Lee et al. (2013) selected the α-wave to calculate the correlation between subjective evaluations of passenger car sounds and their results indicate that the intensity of the correlation between the cerebral α-wave and subjective evaluations can be determined based on the size of the correlation. Nishimura and Mitsukura (2013) put forward a group method of data handling (GMDH) to analyze the sound quality of EERs utilizing neural networks. Compared with the result efficiency of the principal component analysis (PCA), the GMDH neural network resulted in a higher recognition of the target sound quality. The above studies showed that the distinct physiological response of the human brain to sound stimuli authentically exists.

### Contribution

It is difficult to distinguish automobile sounds with similar semantics by means of traditional subjective evaluations. In contrast to the application of EEG signals for emotion recognition, the study of automobile sound quality based on EEG is in infancy, the relationship between EEG feature signals and automobile sound quality is still unclear, and there is less relevant literature. However, there are related research studies on actively playing music based on EEG to improve the subjective emotions of people (Bajaj and Pachori, 2015; Kalaganis et al., 2016). Therefore, a method for mapping EEGs and diversified sound quality for decoding automobile sounds is proposed to reveal the feasibility of using EEG signals as a method of automobile sound quality evaluation, which can avoid language description. The study on decoding automobile sound types can lay the foundation of neuroscience for realizing active playback of automobile sounds based on EEGs in the future.

**Figure 1:**
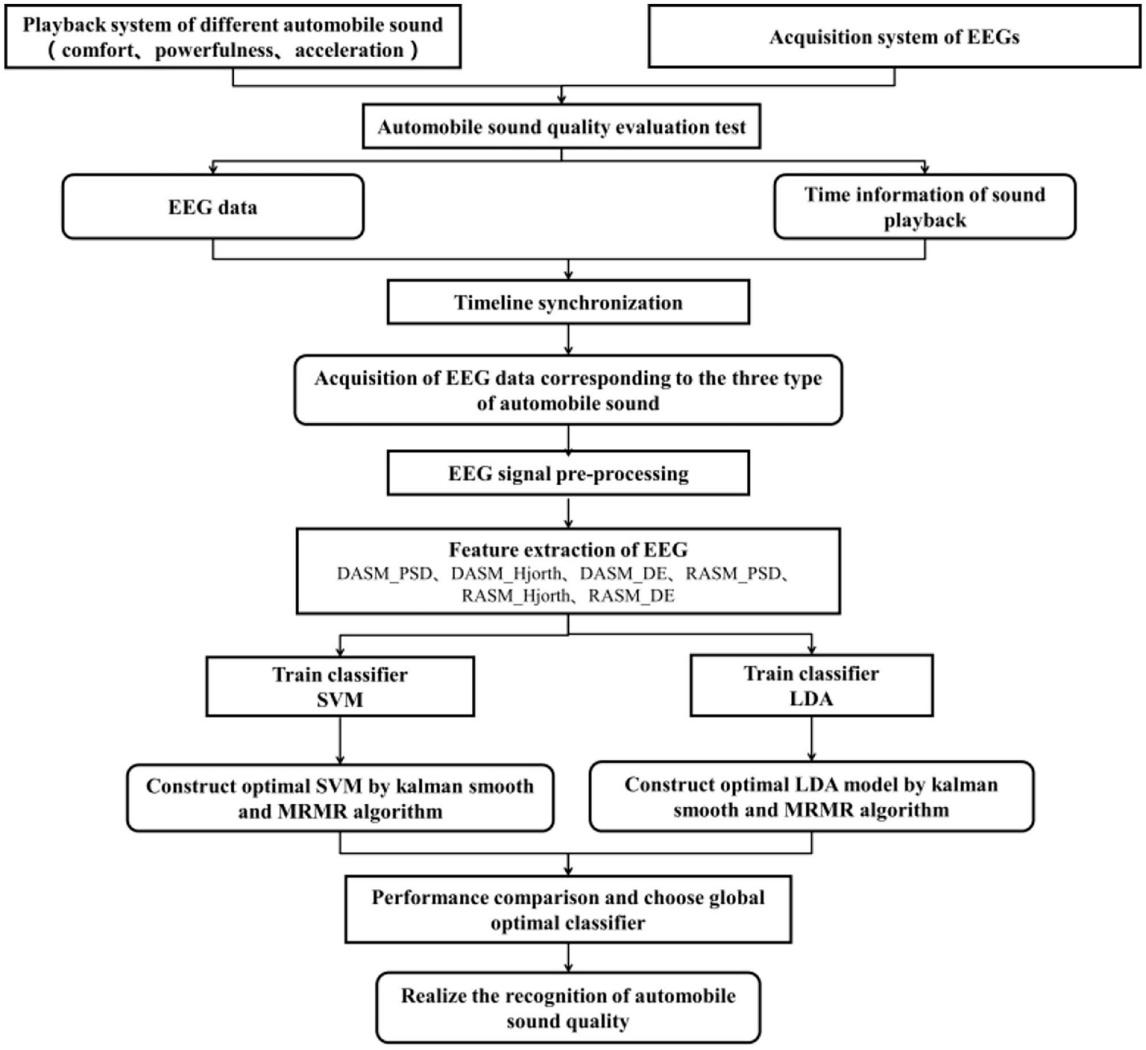
Flow chart for the new evaluation method of car sound quality based on brain signals.

The auditory brain cognition laws refer to the rhythmic activities of the brain under the stimulation of the automobile sound. At present, there are no unified standards for the selection of EEG features, and it requires relevant guidance in selecting EEG features. Thus, changing the law of EEG under the stimulation of automobile sound is studied here, so as to guide the selection of EEG features. By defining three subjective evaluation indices of automobile sound quality (namely, comfort, powerfulness, and acceleration), sounds that matched with the three subjective evaluation indices are collected, The EEGs of the subjects are measured under the stimulation of three automobile sounds, respectively, in a suitable temperature and quiet environment, and the analysis of EEG data contribute to explore the cognition laws of the brain. The differential asymmetry (DASM) and rational asymmetry (RASM) features of subjects are extracted based on cognition laws, and use classification models to identify differences in automobile sound. The flow chart is shown in Figure 1.

### Study Outline

The layout of this study is as follows: the design of the experiment is introduced in section Experiment Design. Section Methodology systematically describes the analysis methods of brain signal feature extraction, selection, and classifier. The results of data analysis are shown in section Experiment Result, including the cognitive laws of the brain under three types of automobile sounds, the use of classification models to compare the recognition accuracy differences of different features, and the optimization of model accuracy using the Kalman smoothing and mRMR algorithm. Section Discussion discusses the results of Section Experiment Result and describes the research significance of this study. Section Conclusions shows the summary and prospects of this study.

## Experiment Design

The three types of automobile acceleration sounds are selected (namely, comfort, powerfulness, and acceleration) as inducing materials for EEG tests. These sounds that cause strong subjective and physiological changes in the subjects are mainly obtained by means of vehicle measurements, online research (such as collect acceleration sound samples of high-end automobile on website sites or from car game software), etc. Table 1 lists the three types of automobile sounds used in the experiment. It is of significance to emphasize that these automobile sounds are divided into three parts, namely, comfort, powerfulness, and acceleration, by 39 engineers with experience in sound quality analysis, and the characteristic distribution of the 39 evaluators is shown in Table 2. The aim of this study is to identify three types of automobile sounds based on EEG signals. Assuming that comfort is −1, powerfulness is 0, and acceleration is 1 here, these data labels make sense when training a classifier.

**Table 1:** Details of the sound clips used in the EEG experiment.

**No**.	**Labels**	**Sound sample sources**	**#Samples**
1	Comfort	obtain the acceleration sound in the car under the WOT of Audi Q5, Audi A8, and FAW Toyota Prada by test	3
2	Powerfulness	obtain the acceleration sound in the car under the WOT of Lexus nx, Alfa Romeo by test; Gets the acceleration audio of Maserati president's car by video website or car game software	3
3	Acceleration	Get comfort car acceleration game simulation audio by video website and car game software	3

**Table 2:** Characteristic distribution of evaluators.

**Category**	**Constituent**	**Quantity**	**Percentage**
Gender	Male	27	70%
	Female	12	30%
Occupation	Teacher	5	13%
	Automotive engineer	24	61%
	Postgraduate	10	26%
Age	20–29 years	26	67%
	30–39 years	5	13%
	40 years or more	8	20%
Driving experience	Yes	30	77%
	No	9	23%

Based on the experimental design and selection of subjects by Zheng and Lu (2015), a total of 15 healthy subjects are recruited, who are different from the 39 engineers. All the subjects included 11 males and four females (aged: 22.4 ± 2.53 years) who are professors or graduate students from the Wuhan University of Technology. They all have experience in automobile sound quality evaluation and ensure their optimal mental health.

**Figure 2:**
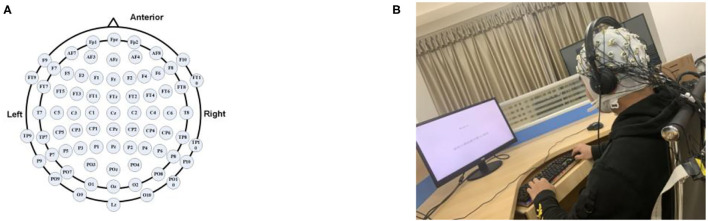
EEG test setup: **(A)** distribution of EEG leads for 62 channels and **(B)** electrode cap test.

Before the experiment started, the test operation procedures and specifications were relayed to all the subjects in advance, and they were instructed to properly wear high-fidelity headphones and press buttons combined with the interface prompts. Making sure that the subjects concentrate on listening to sounds and avoid obvious limb movements during the experiment is of great importance. A 64-channel AgCl electrode cap is used to collect EEG at a sampling rate of 1,000 Hz. The EEG lead distribution and electrode cap test are shown in Figure 2.

**Figure 3:**
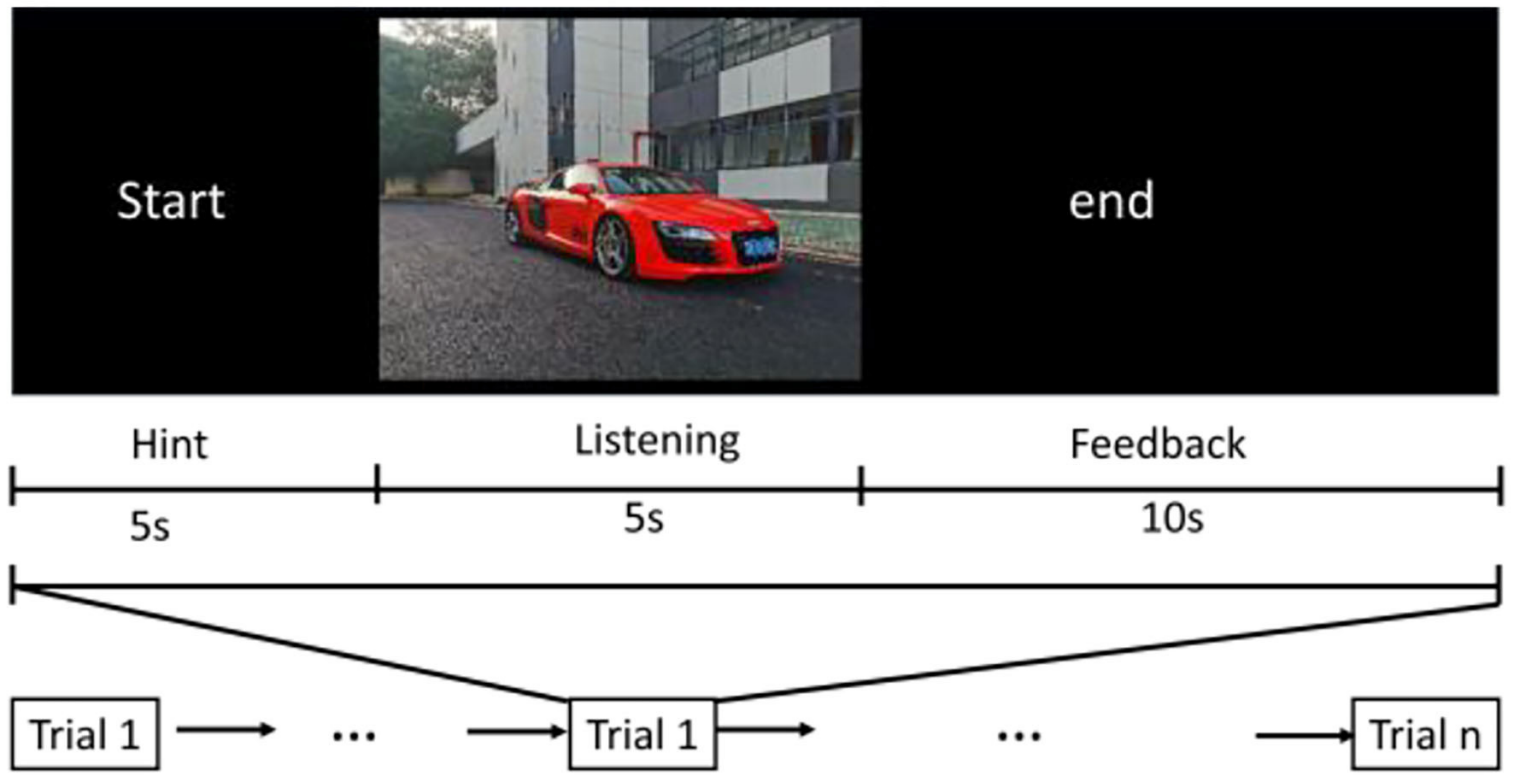
The protocol used in the experiment for sound quality evaluation.

The three automobile sounds in each type are played randomly, and each sound is played 27 times repeatedly. There is a 5 s start prompt before each sound is played, and 10 s rest feedback after playing. A questionnaire format that the computer interface will pop up the type selection item during the 10 s rest feedback period is used, and the subjects judge which type the sound belongs to (namely, comfort, powerfulness, or acceleration). The playback process is shown in Figure 3.

## Methodology

### Feature Extraction

Combining the effective features in the field of emotion recognition, the power spectral density (PSD) (Thammasan et al., 2016), Hjorth (Jorth, 1970), and differential entropy (DE) (García-Martínez et al., 2016) are extracted as the basic EEG features in this study.

The Welch algorithm is used to set a 1-s long rectangular window with an overlap rate of 50% and obtain the PSD corresponding to different frequency bands. The Hjorth parameters, such as activity, mobility, and complexity (Vidaurre et al., 2009; Kaboli et al., 2015) are defined as


(1) $$\begin{array}{l} Activity=\mathrm{var}\left(X\left(t\right)\right) \end{array}$$

(2) $$\begin{array}{l} Mobility=\sqrt{\frac{\mathrm{var}\left(\frac{dX\left(t\right)}{dt}\right)}{\mathrm{var}\left(X\left(t\right)\right)}} \end{array}$$

(3) $$\begin{array}{l} Complexity=\frac{Mobility\left(\frac{dX\left(t\right)}{dt}\right)}{Mobility\left(X\left(t\right)\right)} \end{array}$$

where var denotes the variance of the calculated *X*(*t*) signal.

The DE that satisfies the Gaussian distribution is defined as (García-Martínez et al., 2016).


(4) $$\begin{array}{l} H\left(X\right)=\int_{\text{-}\infty }^{\infty }\frac{\text{1}}{\sqrt{\text{2}\pi \sigma^{\text{2}}}}\exp\frac{{\left(x-\mu \right)}^{2}}{\text{2}\sigma^{\text{2}}}\log\frac{\text{1}}{\sqrt{\text{2}\pi \sigma^{\text{2}}}}\\ \exp\frac{{\left(x-\mu \right)}^{2}}{\text{2}\sigma^{\text{2}}}dx=\frac{1}{2}\log2\pi e\sigma^{\text{2}} \end{array}$$

where *X* means a continuous source, Gaussian distribution satisfies *N*(μ, σ^2^), and π and *e* are a constant.

There are also several pieces of evidence that asymmetry features can well represent the cognitive laws of the human brain (Zheng et al., 2017). In this study, the DASM and RASM of 26 pairs of asymmetric electrodes are calculated, and there are six type features, which are expressed as


(5) $$\begin{array}{l} DASM\text{\_}PSD=PSD\left(X_{\text{left}})-\text{PSD}(X_{right}\right) \end{array}$$

(6) $$\begin{array}{l} DASM\text{\_}Hjorth=Hjorth\left(X_{\text{left}})-Hjorth(X_{right}\right) \end{array}$$

(7) $$\begin{array}{l} DASM\text{\_}DE=DE\left(X_{\text{left}})-\text{DE}(X_{right}\right) \end{array}$$

and


(8) $$\begin{array}{l} RASM\text{\_}PSD=PSD\left(X_{\text{left}})/\text{PSD}(X_{right}\right) \end{array}$$

(9) $$\begin{array}{l} RASM\text{\_}Hjorth=Hjorth\left(X_{\text{left}})/Hjorth(X_{right}\right) \end{array}$$

(10) $$\begin{array}{l} RASM\text{\_}DE=DE\left(X_{\text{left}})/\text{DE}(X_{right}\right) \end{array}$$

**Figure 4:**
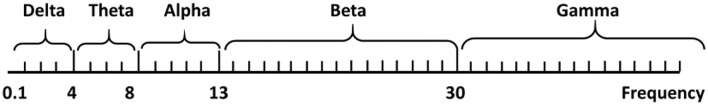
Distribution map of EEG rhythm frequency band.

The frequency is divided into five segments based on the EEG rhythm, as shown in Figure 4. The dimensions of DASM_PSD, DASM_Hjorth, DASM_DE, RASM_PSD, RASM_Hjorth, and RASM_DE are 130 (26 electrodes^*^5 rhythms), 390 (26 electrodes^*^ 5^*^ 3 rhythms), 130 (26 electrodes^*^ 5 rhythms), 130 (26 electrodes^*^ 5 rhythms), 390 (27 electrodes^*^ 5 ^*^3 rhythms), and 130 (27 electrodes^*^5 rhythms), respectively.

### Feature Selection

Herein, the Kalman smoothing algorithm is used to filter out EEG components that are not associated with sounds. The purpose of Kalman smoothing is to calculate the smoothed value of the system state *X*_*k*_ at moment *k* after obtaining all observations up to time *T* (Cheng Y and, 2018), smoothing formula is expressed as


(11) $$\begin{array}{l} p\left(X_{k}|{\text{y}}_{1\;:\;T}\right)=N\left(X_{k}|m_{k}^{8},P_{k}^{8}\right) \end{array}$$

where *T* > *k*, y_1 : *T*_ denotes all observations in the 1~*T* time period and *N*(*X*|μ, σ) denotes the random variable *X* satisfying a Gaussian distribution with mean μ and variance σ. *T* times forward recursion is completed from the initial time 1 to the time *T*, and then perform *T* times backward recursion from the time *T* to complete the Kalman smoothing process. The forward recursion process is Kalman filtering, and the state estimate *m*_*T*_ and covariance matrix *P*_*T*_ at the last time *T* obtained by the forward recursion are the initial state estimate $m_{T}^{8}$ and covariance matrix $P_{T}^{8}$ of the backward recursion process, namely, $m_{T}\;=\;m_{T}^{8},P_{T}\;=\;P_{T}^{8}$.

In addition, the most common problem that is “curse of dimensionality” for pattern recognitions leads to the rapid increase in computation with the increase in feature dimensions (Zheng et al., 2017). It is necessary to select EEG features after smoothing the EEG data with the target of avoiding feature redundancy, and the principal component analysis (PCA) and minimal redundancy maximal relevance (mRMR) algorithm are compared in this study.

The original domain information cannot be preserved by means of the PCA (Nakanishi et al., 2011). Hence, the mRMR algorithm is introduced to select a feature subset from EEG data here. The mRMR algorithm finds a set of features in the original feature set that is strongly correlated with the final output result (Max-Relevance), but the smallest correlation between the features (Min-Redundancy) (Peng et al., 2005). “Max-Relevance” and “Min-Redundancy” are defined as


(12) $$\begin{array}{l} \max D\left(S,c\right),D=\frac{1}{\left|S\right|}\underset{x_{i}\in S}{\sum }I\left(x_{i};c\right) \end{array}$$

(13) $$\begin{array}{l} \min R\left(S\right),R=\frac{1}{{\left|S\right|}^{2}}\underset{x_{i},x_{i}\in S}{\sum }I\left(x_{i},x_{j}\right) \end{array}$$

Combining “Max-Relevance” D with “Min-Redundancy” R, we define Φ(*D, R*) as


(14) $$\begin{array}{l} \max\Phi \left(D,R\right),\Phi =D-R \end{array}$$

The approximate optimal solution can be obtained by the incremental search method, and the feature is selected by maximizing Φ(*D, R*).

### Classifier

The reasonable design of the classifier affects the final result (Ackermann et al., 2016; Jenke et al., 2017; Hernández et al., 2018), and the linear discriminant analysis (LDA) and support vector machine (SVM) are the most common and effective classifiers. Thus, the performance differences between the LDA and SVM models are compared in this study.

The common basic idea of LDA classification assumes that every type of sample data can conform to the Gauss distribution. While a new sample arrives, it can be projected to bring their projected sample features into Gauss distribution probability density function, and then calculate its category corresponding to the peak probability.

The core idea of SVM is to find an optimal hyperplane to achieve the classification effect, and the corresponding decision function is


(15) $$\begin{array}{l} f\left(x\right)\text{= sgn(}\overset{m}{\underset{i=1}{\sum }}\alpha_{i}y_{i}K\left(x_{i},x\right)+b) \end{array}$$

where *x*_*i*_ represents the characteristics of the i-th sample, *y*_*i*_ represents the category of the i-th sample, and α_*i*_ the *b* are the calculation parameters in the SVM optimization process. The mostly used kernel function for EEG signals is the radial basis function (RBF), and the formula is as follows:


(16) $$\begin{array}{l} K\left(x_{i},x\right)={\text{e}}^{-\frac{{\left||x-x_{i}|\right|}^{2}}{2\alpha^{2}}} \end{array}$$

**Figure 5:**
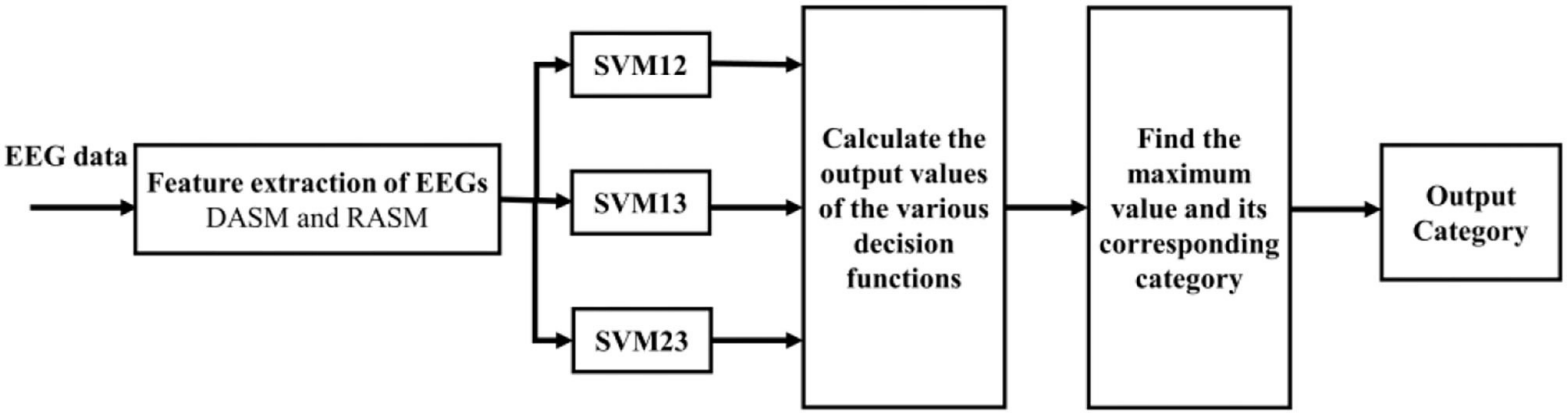
Classification flowchart of SVM.

A “one-to-one” method was used to solve the problems of multi-classification, in which n types of training data are combined in pairs to construct n (n-1)/2 SVM. In this study, the recognition of three types of automobile sound quality is transformed into three two-classification problems. The two important SVM parameters [namely, penalty coefficient (C) and gamma] are tuned by simulation to obtain the optimal SVM model. The three sets of decision function judgment values are output, and the category with the largest judgment value is the output category of sound, namely, majority voting (Ang et al., 2012). The entire classification process is shown in Figure 5.

## Experiment Result

Since the signal-to-noise ratio of EEGs is low, the original data that contain a large number of external interference noises and artifacts are necessarily preprocessed; thus, pure EEG data are extracted with the EEGLAB toolbox, mainly including EEGs (0.1–100 Hz) are captured by means of a band-pass filter, the interference band of 50 Hz is eliminated by a notch filter, the sampling rate is reset to 200 Hz, the artifacts are removed by the method of Independent Component Correlation Algorithm (ICA) and so on.

The data set input to the classification model is N^*^26, where 26 refers to the number of channel pairs, and N is the number of samples. There are a total of 27^*^9^*^5 = 1,215 samples (duration: 1 s) for each subject. After removal of some abnormal data, the number of EEG samples stimulated may be <1,215.

### Cognitive Laws Induced by Automobile Sound

**Figure 6:**
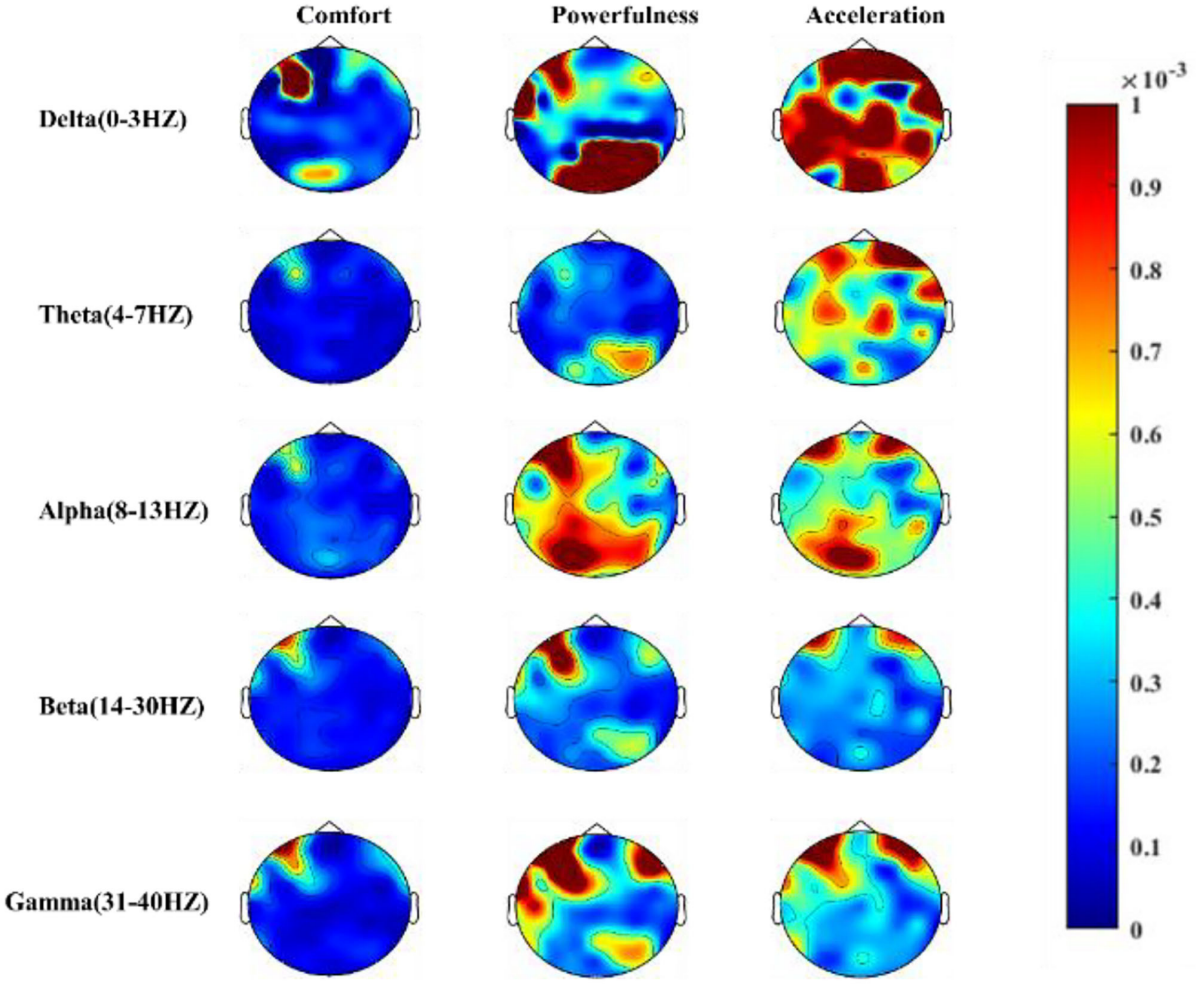
Power topographic maps for the three types of car sounds in five rhythms.

The EEG power topographic map shows the spatial distribution of power of five frequency rhythms, thereby turning complex brain function changes into easy-to-follow graphs. The power topographic maps of five frequency rhythms (δ, θ, α, β, and γ) of the 15 subjects are drawled, as shown in Figure 6.

First, the spectrum power of the five bands under these two kinds of sound stimulation is higher than that of comfort from the perspective of a sense of powerfulness and acceleration. Based on the stimulation of powerful automobile sounds, the energy of the δ rhythm is mainly concentrated in the top and occipital areas of the bottom-right, and the energy is also more prominent in the frontal area of the upper left corner. The θ rhythm is similar to the delta rhythm but lower than δ. The energy of the α rhythm is mainly concentrated in the top area of the lower left and the frontal area of the upper left, and the β rhythm is mainly concentrated in the frontal area of the upper left, and the γ rhythm is symmetrically distributed around the frontal area.

Under the stimulation of acceleration automobile sounds, the δ rhythm energy of the entire brain is more prominent. The energy of θ and α rhythm is symmetrical in the left and right frontal regions, but the energy of θ in the central region is obvious. The energy of α in the left lower occipital region is prominent. The energy distribution of the β and γ rhythms shows a symmetrical distribution in the left and right frontal areas. As for the comfort sounds, the energy of the five frequency rhythms is obvious in the upper left frontal area.

In general, there are clear differences in the frequency band characteristics of EEG rhythm under different quality of sound stimulation.

### Feature Selection

The frequency band energy of the symmetric electrode has a significant difference under the stimulations of diversified automobile sound quality; thus, the symmetrical EEG features are used as input of classifiers in this study. The LDA and SVM are used as classifiers to recognize the three types of automobile sounds, a 5-fold cross-validation scheme is adopted, and the accuracy of the classifier as an evaluation index of classifier performance.

Table 3 shows the mean accuracy of LDA and SVM for symmetrical EEG features (namely, DASM_PSD, DASM_Hjorth, DASM_DE, RASM_PSD, RASM_Hjorth, and RASM_DE) obtained from the five rhythms (δ, θ, α, β, and γ) and the total frequency bands. The LDA average accuracies (%) are 75.01, 84.83, 81.47, 68.50, 83.67, and 80.63 for the six features from the total frequency bands, respectively. For SVM, the average accuracies (%) are 74.83, 86.26, 81.02, 69.11, 85.49, and 81.92. In the total frequency band, the optimal and worst accuracies (%) of the LDA classifier are 86.26 and 69.11, respectively, and for the SVM classifier 84.83 and 68.50, respectively. In the total frequency band, the best and worst accuracy results appear in DASM_DE and RASM_PSD, respectively.

**Table 3:** The mean accuracy rates (%) of LDA and SVM classifiers for different features obtained from separate and total frequency bands.

**Feature**	**Classifier**	**Delta**	**Theta**	**Alpha**	**Beta**	**Gamma**	**Total**
DASM_PSD	LDA	50.63	40.03	44.34	65.17	66.17	75.01
	SVM	59.48	40.68	44.37	66.33	68.27	74.83
DASM_DE	LDA	47.56	46.78	49.33	69.71	83.74	84.83
	SVM	54.98	52.28	52.98	73.35	87.43	86.26
DASM_ Hjorth	LDA	46.83	45.30	51.19	74.05	84.24	81.47
	SVM	49.58	46.45	51.10	75.39	86.08	81.02
RASM_PSD	LDA	42.71	37.59	40.97	62.70	62.89	68.50
	SVM	49.02	39.11	41.78	63.79	64.27	69.11
RASM_DE	LDA	45.58	44.18	47.93	69.76	82.75	83.67
	SVM	51.79	48.94	51.50	73.14	**87.60**	85.49
RASM_ Hjorth	LDA	**40.10**	42.28	48.04	72.45	85.00	80.63
	SVM	44.19	43.91	49.34	74.83	86.85	81.92

*Bold values = highest and lowest accuracy.*

Further, from the classification results of the five rhythms, the LDA classifier has the lowest accuracy with 40.1% in δ rhythms with RASM_Hjorth as the feature. The accuracy up to 87.6% of the SVM classifier is the highest in the γ rhythms with RASM_DE as the feature.

The method of one-factor analysis of variance is used to study the statistical significance of the data, where the results of DE and Hjorth are better than those of PSD, and the difference in classifier performance between LDA and SVM is not apparent (*p* > 0.05). There is a significant difference in classification accuracy (*p* < 0.05) in diverse rhythms, and the accuracies of β and γ bands are significantly better than those of the three rhythms. The classification accuracy of δ, θ, and α is not totally different (*p* = 0.04462).

**Figure 7:**
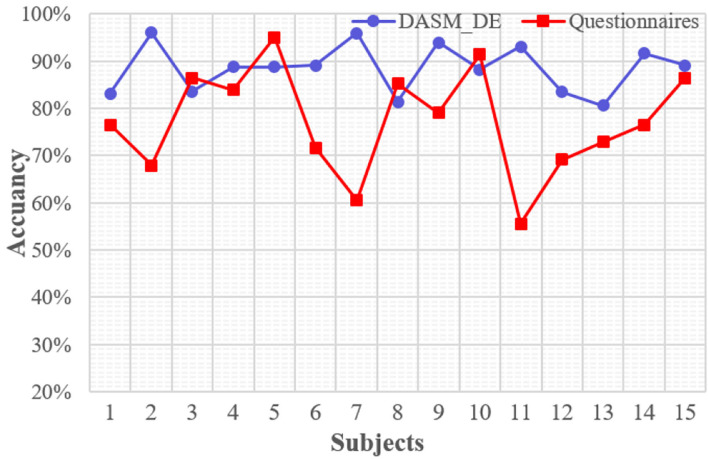
The results of identifying the two types of automobile sounds (namely, powerfulness and acceleration) using SVM with DSAM_DE as the feature and the test subjects in form of a questionnaire.

The powerfulness and acceleration are semantically similar. It is difficult to distinguish the difference based on subjective feelings during the experiment, which is susceptible to lead to confusion. Figure 7 revealed that the semantic similarity recognition effect of automobile sound based on EEG signals is better than that of subjective questionnaire recognition method. Figure 7 shows the results of identifying the two types of automobile sounds (namely, powerfulness and acceleration) using SVM with DSAM_DE as the feature and the test subjects in form of a questionnaire. It is obvious that the accuracy of the questionnaire is lower than machine learning recognition, and the average accuracy of SVM is about 11% higher than the questionnaire. It is worth explaining that the subjective recognition rate of the two other pairwise comparisons (comfort vs. powerfulness and comfort vs. acceleration) is both high, and the average accuracy rate is about 90%, which makes it difficult to reflect the advantages of EEG recognition.

### Optimization of Classifier Accuracy

Firstly, the Kalman smoothing algorithm introduced in section Feature Selection is used here to remove noise that is not related to the desired signal, and the RASM_PSD features of 120 dimensions as inputs, SVM as a classifier. Second, the PCA and mRMR are compared with RASM_Hjorth features of 360 dimensions as inputs and SVM as a classifier.

Table 4 compares the accuracy of the algorithm using Kalman smoothing and without any smoothing algorithm in different rhythms. The accuracy (%) of the unsmoothing method and the Kalman smoothing method in five rhythms is 49.02/68.8, 39.11/60.12, 41.78/62.1, 63.79/84.33, 64.27/85.67, and 69.11/90.36. It is obvious that the accuracy of the Kalman smoothing algorithm method is significantly better than unsmoothing (*p* < 0.05), and the accuracy of the Kalman smoothing method is improved by 19.78% in δ rhythms and 21.4% in γ rhythms. The above results showed that feature smoothing can effectively improve the recognition accuracy.

**Table 4:** The accuracies (%) of unsmoothing and Kalman smoothing method with RASM_PSD features of 120 dimensions as inputs and SVM as a classifier from the total frequency bands.

**State**	**Delta (%)**	**Theta (%)**	**Alpha (%)**	**Beta (%)**	**Gamma (%)**	**Total (%)**
Unsmoothing	49.02	39.11	41.78	63.79	64.27	69.11
Smooth	68.8	60.12	62.1	84.33	85.67	90.36
Difference	19.78	21.01	20.32	20.54	21.4	21.25

**Figure 8:**
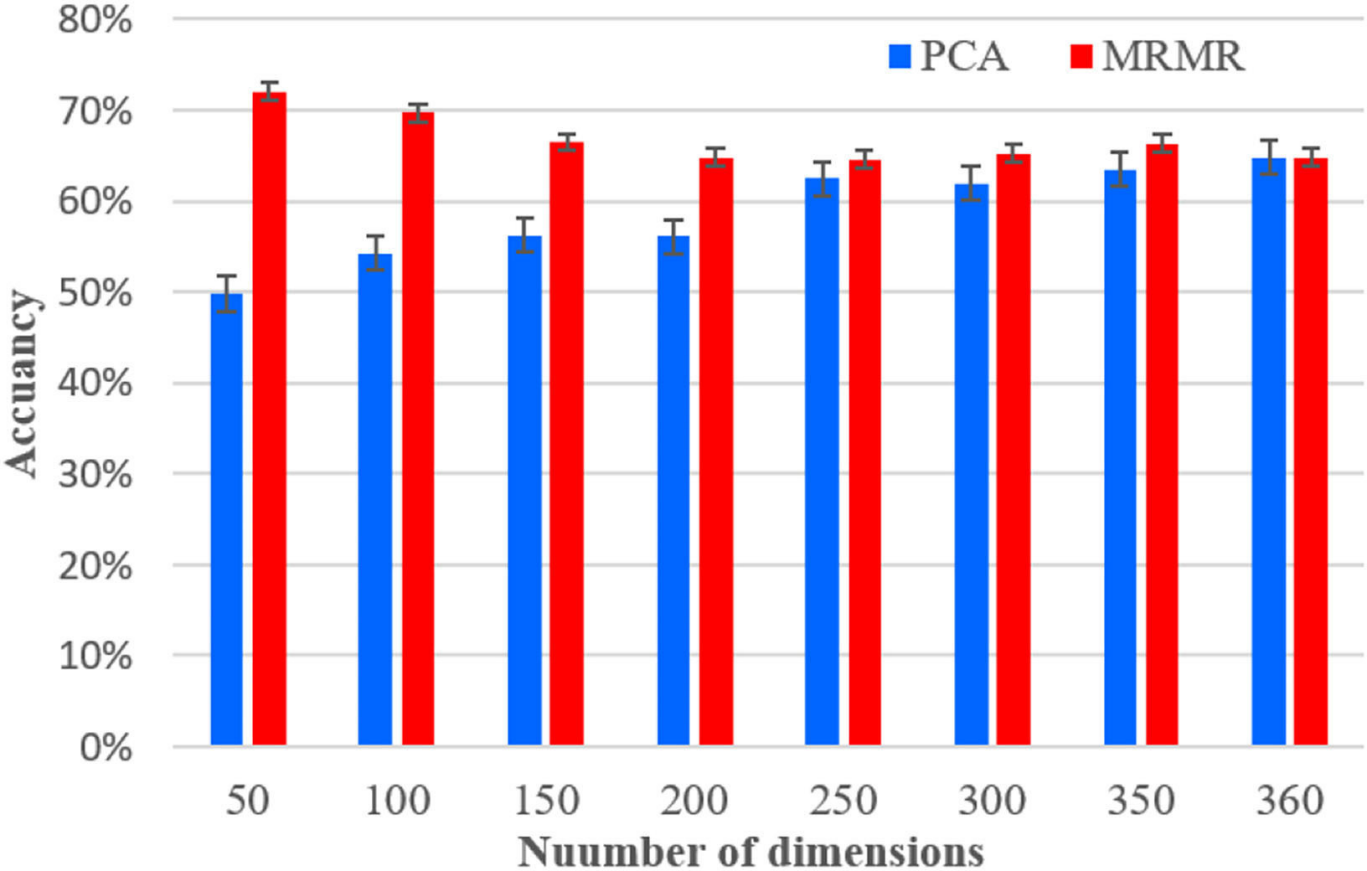
The average accuracies of SVM using RASM_ Hjorth obtained from total frequency bands base on PCA and mRMR for subject 12.

Figure 8 compares the impact of dimension reduction using PCA and MRMR algorithms on model precision performance, in which the dimension of the model is reduced from 350 to 50 dimensions with 50 intervals. It is clear that the usage of the PCA algorithm, which can reduce the dimensionality, does not significantly improve the accuracy. The accuracy rate drops from 64.8 to 49.8% when the dimensionality reduced to 50, and it reaches 62.5% at 250 dimension, which is lower than the original 360 dimension of 1.7%. However, the mRMR algorithm can not only reduce the dimensionality, but also improve the accuracy of the classifier, the accuracy using the mRMR algorithm reached the local maximum (72.00%) at 50 dimension, which is 7.2% higher than the original 360 dimension. Moreover, the accuracy improved significantly when the dimension is 50, 100, and 150, and the dimensionality reduction is not obvious when the dimension is >150.

## Discussion

This study demonstrates the feasibility of EEG-based recognition of the diversified sound quality of the automobile. Several important issues are explored.

Some studies showed that the brain waves in a certain rhythm band are indeed aroused (Lee et al., 2013; Lee and Lee, 2014) under the stimulation of automobile sounds. As shown in Figure 6, there are frequency band differences in brain cognition under the stimulation of different sounds, which is specifically reflected in positive the correlation between EEG energy and sound energy intensity. The recognition of automobile sound quality is improved based on frequency band characteristics, which can well reflect the laws of brain cognition. Some literature has proved that the frontal area is closely related to human brain cognition (Saxe, 2006; Shamay-Tsoory and Aharon-Peretz, 2007), and there is a large proportion of energy in the frontal area under musical stimulation (Sammler et al., 2010; Di and Wu, 2015). Therefore, the results shown in Figure 6 of this study provide further evidence that the cognition laws in the frontal portion of the human brain can indeed be aroused by automobile, so as to guide the selection of EEG features.

The DASM has better classification accuracy than RASM, which is consistent with the conclusion of the literature (Lin et al., 2010). Among the three basic features (PSD, Hjorth, DE), DE has the best classification performance, and it is most suitable for the recognition of automobile sounds. Although the classification accuracy of DASM_DE and DASM_Hjorth is close, the dimension of DASM_DE is 1/3 of DASM_Hjorth. Among the five rhythms, the classification accuracy of the β and γ rhythms is better than the other three rhythms, which proves that the correlation between different sound quality and different rhythms of brain waves is also different. The classification accuracy of the SVM model is slightly better than LDA, but SVM has the advantages of a small number of training sets, fast training speed, and high accuracy. The best accuracy of motion classification (82.29% ± 3.06%) is obtained by SVM, as demonstrated in the literature in both Lin et al. (2010) and Hadjidimitriou and Hadjileontiadis (2012), which are both similar to our study.

The comfortable sound is light and natural, and the sound pressure level is small. On the contrary, the other pairs are powerful, booming, and exciting, and the topographic map corresponding to the comfort as shown in Figure 6 differs significantly from the other two types. For experienced automotive engineers, it is easy to distinguish the sound characteristic difference between comfort and powerfulness (or acceleration), but it is difficult to distinguish the difference between the powerfulness and acceleration sounds. In Figure 7, compared with recognizing sounds based on subjective feelings, using the classification model has higher recognition accuracy based on EEG characteristics. The literature (Nakanishi et al., 2011) verified the difference of EEG between three kinds of acoustic quality by using PCA and FDA in a similar way to this study. In which, the result proved that they can obtain the information that they cannot obtain from questionnaires by EEG. It is possible that the change of subjective emotion is provoked by the stimulation of the automobile sounds. However, it is not yet clear which emotion it is related to and it is the next step in the research.

As discussed in section Feature Selection, the Kalman smoothing algorithm can effectively improve the recognition accuracy and confirm that feature smoothing plays an important role in EEG-based recognition. In Figure 8, it is obvious that the mRMR algorithm is an effective method to optimize the accuracy of recognition, which retains the original information, such as electrode channels and frequency bands, while reducing the complexity of calculations. In the literature (Zheng et al., 2017), the mRMR algorithm was also used to achieve dimensionality reduction for improving recognition accuracy of emotion, which improves the accuracy by 14.41%.

The main contributions of this study to sound quality recognition from EEG can be summarized as follows: (1) an EEG signal acquisition test paradigm is designed based on automobile sounds, which provide experimental guidance for studying the correlation between automobile sounds and EEG signals; (2) it was systematically described the processing process of EEG data from three aspects: feature extraction, feature selection, and pattern recognition and proves that the selection of EEG features, the smoothing and dimensionality reduction of data, and the reasonable design of classifier are crucial for the recognition of sounds; (3) this study confirms that the neural characteristics of the three types of automobile sounds do exist, and the SVM can effectively identify the three types of automobile sounds through the input of the DASM_DE of γ rhythm; and (4) this research takes the brain–computer interface technology as the breakthrough point and introduces the physiological features of EEG to recognize the automobile sound quality innovatively.

## Conclusions

The objective of this research is to investigate the laws of brain cognition under the stimulation of diverse automobile sounds and propose an effective method to identify diversified automobile sounds. The results show that the frequency band features can well reflect the laws of brain cognition, which can effectively realize the recognition of automobile sound quality by constructing asymmetric EEG feature indices and using machine learning models. The DASM_DE of the γ rhythm is used as the input, and the accuracy of automobile sounds reached up to 86.26% by SVM. Also, it proves that the Kalman smoothing and mRMR algorithm can not only improve the recognition accuracy but also reduce the amount of model calculation. In summary, this study proposes a new method of automobile sound quality recognition from the field of brain–computer interface technology.

Future study will include further evaluation of the specific relationship between EEG signals and the inherent characteristics of automobile sounds, proposed indices that can quantify automobile sound quality, and the usage of deep learning algorithms that automatically extract the potential features of EEGs.

## Data Availability Statement

The raw data supporting the conclusions of this article will be made available by the authors, without undue reservation.

## Ethics Statement

The studies involving human participants were reviewed and approved by the Ethical Review Committee of Wuhan University of Technology. The participants provided their written informed consent to participate in the study.

## Author Contributions

ZL and LX designed the data processing schema and wrote the manuscript. CL and TX designed the experiment and were involved in the data collection. LY made a great contribution to the content of the manuscript during the revision process. All authors contributed to the article and approved the submitted version.

## Funding

This work supported by National Natural Science Foundation of China (Grant Nos. 52175111 and 61876137) and Foshan Xianhu Laboratory of the Advanced Energy Science and Technology Guangdong Laboratory (No. XHD2020-003).

## Conflict of Interest

The authors declare that the research was conducted in the absence of any commercial or financial relationships that could be construed as a potential conflict of interest.

## Publisher's Note

All claims expressed in this article are solely those of the authors and do not necessarily represent those of their affiliated organizations, or those of the publisher, the editors and the reviewers. Any product that may be evaluated in this article, or claim that may be made by its manufacturer, is not guaranteed or endorsed by the publisher.
